# Smart Self-Sensing Composite: Piezoelectric and Magnetostrictive FEA Modeling and Experimental Characterization Using Wireless Detection Systems

**DOI:** 10.3390/s20236906

**Published:** 2020-12-03

**Authors:** Relebohile George Qhobosheane, Muthu Ram Prabhu Elenchezhian, Partha Pratim Das, Minhazur Rahman, Monjur Morshed Rabby, Vamsee Vadlamudi, Kenneth Reifsnider, Rassel Raihan

**Affiliations:** 1 Department of Mechanical and Aerospace Engineering, University of Texas at Arlington, Arlington, TX 76019, USA; relebohile.qhobosheane@mavs.uta.edu (R.G.Q.); muthuramprabhu.elenchezhian@uta.edu (M.R.P.E.); parthapratim.das@mavs.uta.edu (P.P.D.); minhazur.rahman3@uta.edu (M.R.); monjurmorshed.rabby@mavs.uta.edu (M.M.R.); kenneth.reifsnider@uta.edu (K.R.); 2 Institute of Predictive Performance and Methodologies, University of Texas at Arlington Research Institute, Fort Worth, TX 76118, USA; vamsee.vadlamudi@uta.edu

**Keywords:** magnetostriction, wireless sensors, piezoelectric, fiber-reinforced composites, planar coils, magnetization

## Abstract

This research work focuses on the development of a piezoelectric magnetostrictive smart composite with advanced sensing capability. The composite piezoelectric property is achieved from the dispersion of single-walled carbon nanotubes (SWCNTs) and the magnetostrictive property from Terfenol-D nanoparticles. Finite element analysis (FEA) is used to examine the feasibility of modelling the piezoelectric (change in electric field) and magnetostrictive (change in magnetic field) self-sensing responses in the presence of applied stress. The numerical work was coupled with a series of mechanical tests to characterize the piezoelectric response, magnetostriction response and mechanical strength. Tensile tests of the composite samples manufactured as is (virgin), samples with SWCNTs, samples with Terfenol-D nanoparticles and samples with both SWCNTs and Terfenol-D nanoparticles were conducted. It was observed that an increase in volume fraction of Terfenol-d nanoparticles increases the change in magnetization, therefore increasing voltage response up to the point of saturation. The optimum change in amplitude was observed with 0.35% volume fraction of Terfenol-D nanoparticles. A constant ratio of SWCNTs was maintained, and maximum change in electrical resistance was at 7.4%. Fracture toughness for the samples with all nanoparticles was explored, and the results showed improved resistance to crack propagation.

## 1. Introduction

**Figure 1:**
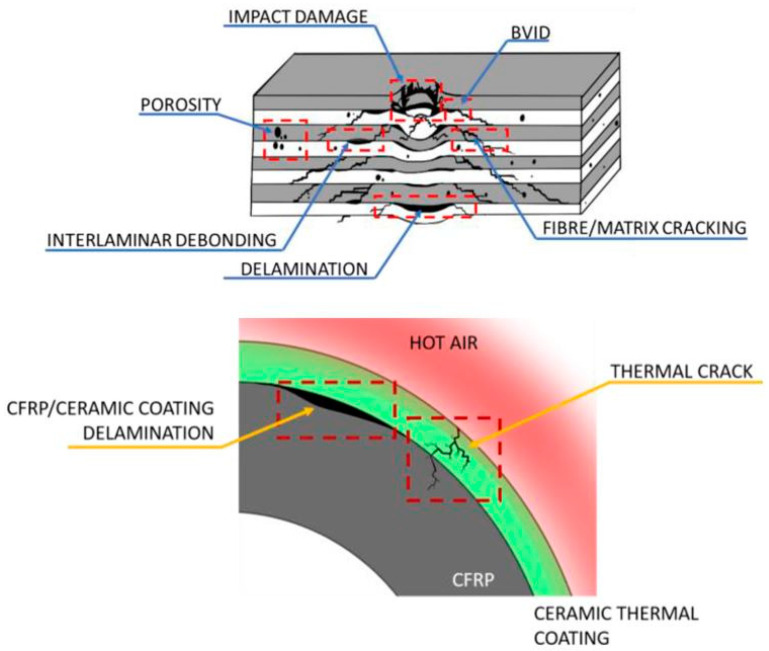
Composite structure defects leading to the need for advanced structural health monitoring methods [7].

The development of smart self-sensing composite materials has been prominent in recent years. This is due to the need for advanced nondestructive structural health monitoring methods [1]. Composite materials during fabrication and their operation are exposed to different stress conditions [2,3]. These applied stresses induce various types of composite damage modes. The damage can be microscopic defects, which are sometimes negligible but can affect the strength of the overall composites, or they can be macroscopic, which are due to the composite structural features [2,4]. During the fabrication process of composite materials, minor voids may be generated between the matrix and fibers, resulting in increased composite porosity. Voids in close proximity within a composite may lead to the generation of cracks [5]. Delamination is another defect type that may results due to different loading conditions on the composite sample [6]. Propagation of minor defects such as voids and matrix cracks can also lead to delamination within a composite sample [7]. These types of defects, including barely visible impact damage (BVID), are generalized in the schematic shown in Figure 1 for carbon fiber-reinforced polymer (CFRP) composites. The matrix and fiber are represented by the light grey and white colors, respectively, while the composite is presented by the grey color. The green color represents the ceramic thermal coating, and red represents hot air.

### Background

The use of piezoelectric materials in smart composites development has shown great strides in recent years. In the manufacturing of composites, carbon nanotubes have attracted the most attention. Numerous studies have focused on this, including the work of Denish Kumar [8], who worked on the characterization of glass fiber composites with multi-walled carbon nanotubes at high temperatures. Their work showed that 0.1% multiwalled carbon nanotubes (MWCNT) in glass fiber composites contribute to an increase in flexural strength and modulus of the composite. The focus of this work is on the change in piezoelectric response of the carbon nanotubes (CNTs) within the composite. The presence of CNTs within composites contributes to the improvement of the piezoelectric response. Fan Yang [9] explored the effect of CNTs added using different coating methods on engineered cementitious composites. The application of mechanical stress showed a variation in electrical resistance sensitivity in these composites. Variation of CNT volume fraction contribution to the change in electrical resistance in composites was also explored in [10]. This study showed that the change in CNT volume fraction directly contributes to the electrical resistance change, and, therefore, an optimum composites constituent ratio was confirmed for different loading studies [10]. Magnetostrictive materials have also been of interest in composite research. Sputtering of a specific combination of a rare earth magnetostrictive materials in amorphous alloys can yield a material with a high magnetostriction. Iron–cobalt (FeCo) alloy was used in the development of smart composites for sensing applications. The drawn FeCo fibers mixed with diglycidyl ether of Bisphenol-F and a polyamine curing agent for matrix results in a FeCo fiber-reinforced composite which is sensitive to bias magnetic field changes [11]. Further developments of magnetostrictive composite materials are explored in the development of whisker sensors [12]. Alfenol and Galfenol alloys are melted with other metals, cold rolled to the required thickness and annealed to thin sheet samples [12] that can be used in magnetostrictive materials applications. The magnetostrictive materials were wrapped with plain weave carbon fiber and epoxy. The fabricated smart composites show variations in magnetization as result of applied stress [12]. Numerous studies [13,14] have contributed to this area in developing smart composites. In this work, Terfenol-D magnetostrictive material was used to develop a smart self-sensing fiber-reinforced composite.

## 2. Smart Self-Sensing Composite FEA

**Figure 2:**
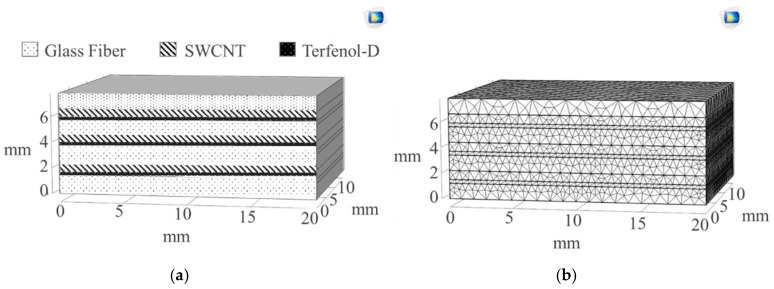
Smart self-sensing composite (**a**) labeled layer model and (**b**) model after running mesh.

Modelling of the smart self-sensing composite used in this work was carried out in COMSOL Multiphysics^®^. A 3D model was developed for a composite with four glass fiber layers and three interfacial regions consisting of Terfenol-D particles and single-walled carbon nanotubes (SWCNTs), as shown in Figure 2. The materials used in this system with their properties include those given in Table 1.

### 2.1. Geometry and Boundary Conditions

The model aspect ratio was 4:5 with a length of 10 mm for all layers, SWCNT layer thickness of 0.7 mm as per the diameter of carbon nanotubes selected, Terfenol-D layer thickness of 0.3 mm as per the diameter of the particles, and glass fiber-reinforced polymer composite (GFRP) thickness of l.2 mm. Each layer was assigned to its individual material property. The initial composite model is therefore shown in Figure 2. Physics-controlled mesh sequence type was followed for this model, maintaining fine element size [15].

### 2.2. Multi-Physics Model

A solid mechanics model was used for quasi-static loading, excluding second-order time derivatives and mass effect of the composite for an efficient solution. Even though Lagrange displacement field has better accuracy, quadratic serendipity analysis [16] was selected for the model shape functions to control the displacement field of the composite model. This solid mechanics model solves the equations of motion for the composite system and its predefined material properties. The stress due to the boundary load force $F_{v}$ in the y-direction coupled with density is governed by Newton’s second equation [15]:

(1) $$\nabla \cdot \sigma +F_{v}=\rho \ddot{u}$$

where σ is the stress due to the boundary load, ρ the density, and $\ddot{u}$ as the acceleration vector. The static composite model was conducted through a stationary solver, therefore making the right side zero. The composite model elasticity tensor was built from Lame constants [17]. Therefore, the relation between the displacement and resulting strains for the smart self-sensing composite is given by

(2) $$\varepsilon =\frac{1}{2}\left[\nabla u+{\left(\nabla u\right)}^{T}\right]$$

**Figure 3:**
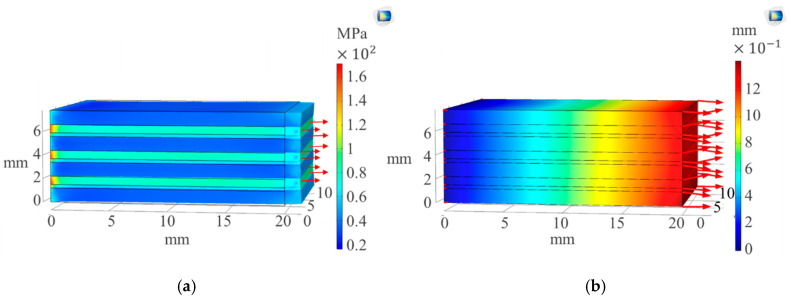
(**a**) Stress (MPa) concentration during loading and (**b**) the displacement (mm) of the composite.

The composite system was fixed on the z axis to replicate the tensile tests conducted in quasi static experiments and a boundary load applied on one end, as shown by arrows in Figure 3a. Stress distribution during loading is shown in Figure 3a with high concentrations on the support and the composite displacement shown in Figure 3b.

**Figure 4:**
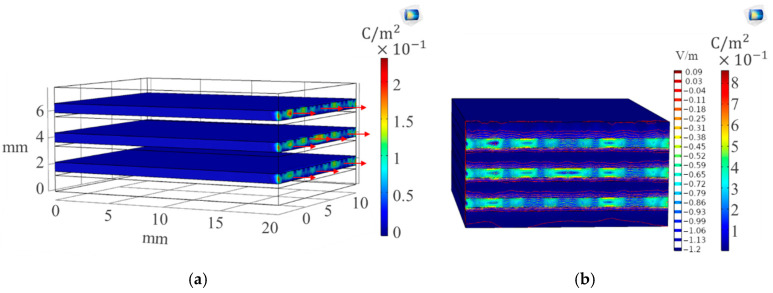
The (**a**) piezoelectric layer polarization (C/m^2^) and (**b**) electrical field (V/m) layer response.

The analysis of Von Mises stress [18] by COMSOL Multiphysics^®^ is shown in Figure 3a, where we have response of the composite sample to applied stress focused more on the fixed end. The free end showed a change in length as the boundary load was applied. The displacement concentration and direction on Figure 3b were high on the free end. The composite modeled entailed within it a piezoelectric characterization problem. The piezoelectricity of the sample was dependent on the amount of strain applied leading to electrical polarization. SWCNT layer physics shown in Figure 4 were modeled by coupling solid mechanics properties with electrostatic physics. COMSOL Multiphysics^®^ relates the strain due to the applied boundary load to the polarization of the piezoelectric material by solving the equations [18].

(3) $$\varepsilon =s_{E}\sigma +d^{T}E$$

(4) $$D=d\sigma +\varepsilon_{T}E$$

where ε represents the strain applied on the composite sample, σ is the stress due to the boundary load, E is the electrical field, D is the displacement field, $\varepsilon_{T}$ is material permittivity, d represents the coupling properties, and $s_{E}$ is the material compliance. The applied boundary load on one end of the composite results in applied stress in three directions with corresponding electrical fields due to the piezoelectric material [19]. The orientation of the SWCNT piezoelectric layer is also dependent on the defined material properties.

Electrical field change in the piezoelectric material was observed more on the free end of the composite sample than for all other directions. This illustrated the effect of change in strain on the material electrical resistance. At initial strains, a highly sensitive response in electric potential was observed up to a peak of 19 C/mm^2^. A reduction in polarization was noticed with increase in strains from this point and a further nonlinear increase in the electrical field. The SWCNT layer was attached to both the glass fiber-reinforced composite and to the Terfenol-D magnetostrictive layer, and assembly formed. The behavior of the Terfenol-D layer was characterized by coupling the solid mechanics model with magnetic fields to model the magnetostrictive response. The behavior of the nonlinear magnetic field H was modeled linearly in COMSOL Multiphysics^®^ to enable relation to the strain, stress and magnetic flux by strain–magnetization relationships. Magnetization [20] was formulated using both stress and magnetic field:

(5) $$\frac{\mathrm{dM}}{\mathrm{dt}}=\left(\frac{\partial M}{\partial H}\right)\frac{\mathrm{dH}}{\mathrm{dt}}+\left(\frac{\partial M}{\partial \sigma }\right)\frac{d\sigma }{\mathrm{dt}}$$

**Figure 5:**
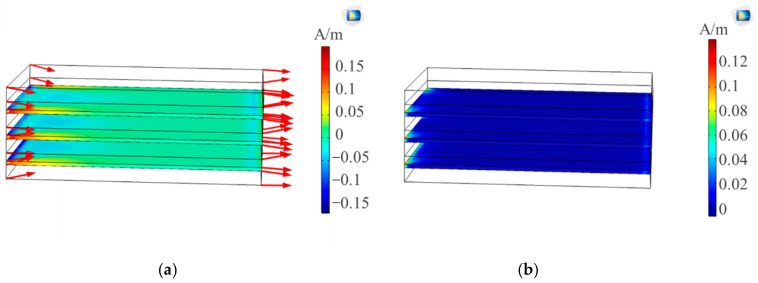
Composite model (**a**) magnetic field and strain direction; (**b**) change in magnetization (A/m).

The Villari effect of the magnetostrictive material layer is characterized by the magnetization change due to the magnetic field. The magnetic field H applied to the composite system contributed to the magnetization through the field derivative dH/dt. Characterization of the magnetic induction [21] due to loading of the model was characterized by the resulting stress and magnetostrictive constant with the permeability at constant stress. The strains generated within the magnetostrictive material contributed to the change in magnetic field. Magnetization caused by the magnetostriction [22] of the cubic material was characterized by λ [23], which represents the scalar magnetostriction measured along a given direction:

(6) $$\lambda =\frac{3}{2}\frac{\lambda_{100}}{M_{s}^{2}}\left[M_{1}^{2}\left(\beta_{1}^{2}-\frac{1}{3}\right)+M_{2}^{2}\left(\beta_{2}^{2}-\frac{1}{3}\right)+M_{3}^{2}\left(\beta_{3}^{2}-\frac{1}{3}\right)\right]\;+3\frac{\lambda_{111}}{M_{s}^{2}}\left(M_{1}M_{2}\beta_{1}\beta_{2}+M_{2}M_{3}\beta_{2}\beta_{3}+M_{1}M_{3}\beta_{1}\beta_{3}\right)$$

where β represents the directional cosines, $\left[\lambda_{100},\;\lambda_{111}\right]$ represents the magnetostrictive coefficients, and $M_{s}$ represents the saturation magnetization; therefore, the effective field of the magnetostrictive model was given as

(7) $$H_{\mathrm{eff}}=H+\frac{3}{{µ}_{0}M_{s}^{2}}\left[\lambda_{100}S_{\mathrm{ed}}+\left(\lambda_{111}-\lambda_{100}\right)\underset{i\neq j}{\sum }{\left(S_{\mathrm{ed}}\right)}_{\mathrm{ij}}\left({℮}_{i}⊗{℮}_{j}\right)\right]M$$

where $S_{\mathrm{ed}}$ is the deviatoric stress tensor and ${µ}_{0}$ is the magnetic permeability of free space. This change in magnetic field in the direction of strains is reflected in Figure 5a and the resultant magnetization in Figure 5b. The magnetostrictive Terfenol-D layer turned out to be highly sensitive to the generation of strains resulting in magnetic fields in the same direction. This change was observed at both the free edge and fixed supported edge with a higher tendency towards the fixed supported edge to show higher stresses.

**Figure 6:**
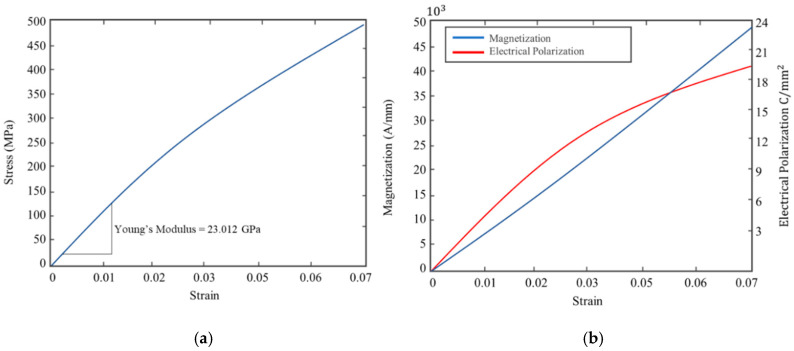
(**a**) Stress–strain response of the smart self-sensing composite; (**b**) magnetization and electric polarization or the composite with change in strain.

Coupling of solid mechanics, electrostatics and magnetic fields physics for the composite system enabled the relation of the composite strain to both magnetization change and electrical polarization, as shown in Figure 6. The stress–strain relationship of the smart self-sensing composite is shown in Figure 6a and magnetostrictive response with piezoelectric response in Figure 6b.

The overall elastic modulus of the composite with a combination of the three constituents, GFRP, Terfenol-D and SWCNT, was 23.012 GPa. A linear change in magnetization was observed with the increase in strains, as shown in Figure 6b. Constant electrical polarization was observed up to a strain of 0.028, where a slight decrease in electrical polarization gradient was noted. This results are compared with experimental data in the next sections. This was the formulation of the solid mechanics and physics used in the application of stresses to generate strain on the smart self-sensing composite used in the present work. This included both magnetostriction and piezoelectric property characterization of the composite.

## 3. Experimental Methodology

The development of the piezoelectric magnetostrictive self-sensing composites in this work was completed in two steps. First was the design and fabrication of the composite, detailing the constituents in this composite to give it its piezoelectric and magnetostrictive properties. The next step was the development of the detection method used. To experimentally characterize the smart self-sensing composite piezoelectric and magnetostrictive response, an advanced magnetic flux density and electrical change detection device was developed. The composite specimens were run through a series of mechanical tests to explore their self-sensing capabilities and limitations for further research and applications.

### 3.1. Piezoelectric Solution

**Figure 7:**
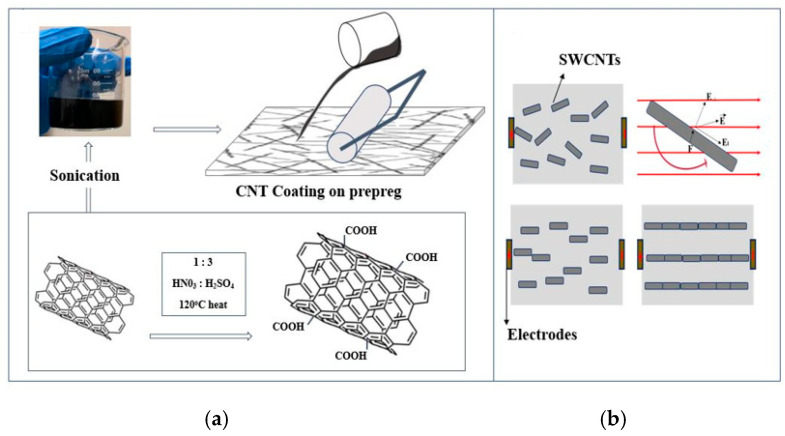
Steps followed in (**a**) the fabrication of SWCNT fiber composite and (**b**) alignment of SWCNTs.

The fabrication of smart self-sensing composites in this work followed a unique approach. Terfenol-d nanoparticles were used for sensing material and carbon nanotubes for the improvement of the composite mechanical properties. The preparation of SWCNTs in this work also followed a unique approach. Single-walled nanotube from Sigma Aldridge with an inner diameter of 0.7–1.1 nm were used. These SWCNT had chirality of 7.6, and the materials used for preparation were nitric acid (HNO_3_), sulfuric acid (H_2_SO_4_) and isopropanol. The properties of SWCNTs due to their van der Waal forces lead to their agglomeration. For the purpose of this work, agglomeration of SWCNTs was evaded by functionalizing the SWCNTs in acid. Exohedral functionalization was followed by dispersing 50 mg of SWCNTs in 150 mL of 1:3 HNO_3_: H_2_SO_4_ solution under 120 °C heat and stirred at 500 rpm. After acid treatment of this SWCNTs, deionized (DI) water was used to clean the material thoroughly. The next step was ultrasonication in isopropanol of the SWCNTs for 1 h. Hydroxyl and carboxyl groups that can interact with oxygen containing groups in glass fiber prepreg were generated from chemical oxidation. The ultrasonicated solution was then dispersed on prepreg fibers for composite fabrication. These steps are summarized in Figure 7a. The electrical field was then applied to the prepreg with SWCNTs, as shown in Figure 7, to alter their orientation. SWCNTs rotate and shift to form a head-to-head contact moving towards the negative electrode, therefore forming developing and aligned networks.

### 3.2. Magnetostrictive Solution

**Figure 8:**
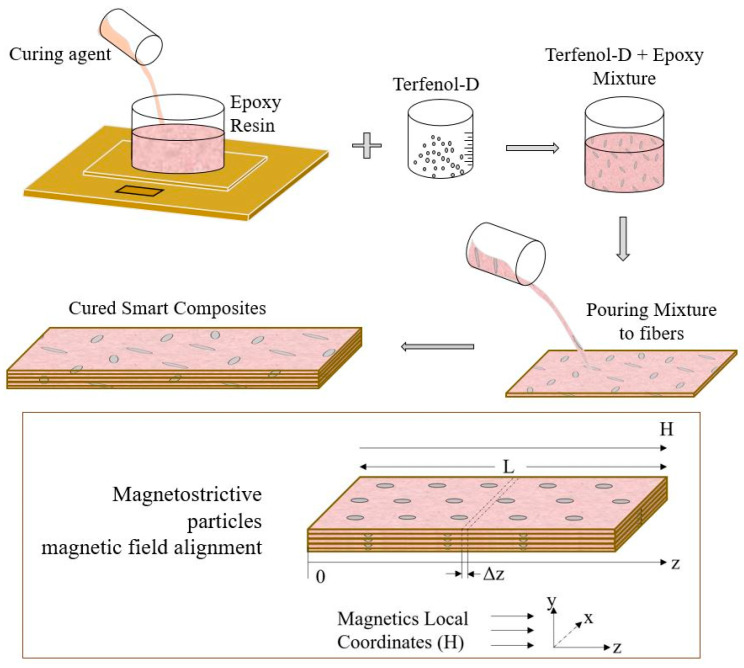
Fabrication procedure of smart self-sensing composites.

To develop a self-sensing composite with magnetostrictive properties, Terfenol-D particles were added as part of the final composites. To do this, the epoxy diglycidyl ether of Bisphenol-F and a polyamine curing agent matrix were mixed with Terfenol-D particles with a size of 0–300 µm. Slow mixing using a wooden stirrer was performed, followed by light brushing of the solution onto glass fiber prepregs. Distribution of these nanoparticles throughout the fibers was a quick step. After 24 h of refrigeration, the plies were then laid up for fabrication, and the fabricated samples passed through a magnetic field H in Tesla to align the magnetostrictive particles field. The fabrication procedure is shown in Figure 8.

The reason these nanoparticles were selected is that Terfenol-D magnetostrictive alloy has a high magnetostrictive property in the range of 800–1200 ppm and a Young’s modulus of 50–90 GPa. The presence of Terfenol-D particles within the composite contributed to the overall composite magnetic, electric, mechanical and thermal properties. This was therefore bound to change the composite stress–strain properties and damage modes within the system.

### 3.3. Compression Molding

Glass fiber unidirectional prepreg was selected for the composite used in this work. For all the samples tested, four plies of prepreg were cut into dimensions of 220 by 220 mm. All prepreg substrates were pre-weighted before fabrication. The first set of samples were of glass fiber-reinforced composite; the second set was of prepregs brush coated with the piezoelectric solution; the third set of samples were brush coated with the magnetostrictive solution; the last samples had both piezoelectric and magnetostrictive solution coatings. Two types of layups were conducted in this work: the first set of samples’ layup orientation was 0° for all samples; the second set stacking orientation was a 45°, −45°, −45°, 45° layup. The next step was placing the mold with the layup inside the compression molding chamber and moving it up towards the top mold. Before closing the chamber, the fabrication sequence was set with maximum compression pressure of 4 tons and a temperature of 275 °C. The chamber was then closed, and preheating started. The heating cycle for the glass fiber-reinforced composites was set for 48 min, in addition to a cooling cycle for 48 min with a cure time of 90 min. The mold and release layers were then peeled off the fabricated samples. The fabricated composite samples were then cut into specimens of dimensions 180 by 22 mm and taken for mechanical testing.

### 3.4. Detection System

**Figure 9:**
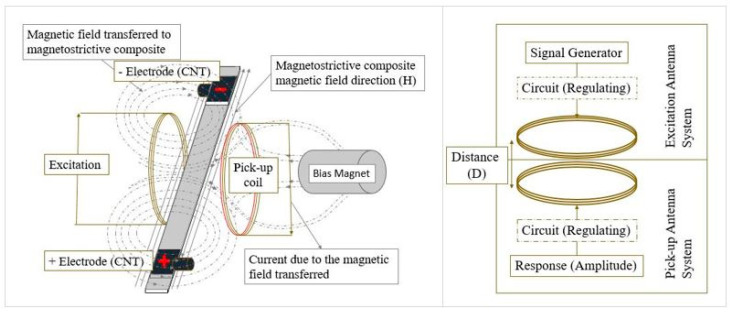
Working system of the designed magnetostrictive self-sensing smart composites detection system.

The detection system used in this work for wireless detection of signals generated by the self-sensing smart magnetostrictive composite was based on the working principle of the transmitter and receiver antennas. Coupling of the two antenna coils with optimal detection response depends on various parameters, which include antenna coils design, coils dimensions, number of turns, and excitation signal type, amongst other parameters. The excitation and pickup coils in this work were of 0.025 mm^2^ area and 1000 turns. Excitation frequency was at 50 kHz, and the magnetic field of 0.384 T was applied around the sensor system. The working principle of the detection system is based on electromagnetic coupling between the antenna coils and change in magnetization around the smart self-sensing composite due to the application of loading. Incident stresses/strains on the magnetostrictive self-sensing composite will give a reaction change in the magnetic field around the composite due to its Villari effect. This change in magnetization is used to characterize the generation of defects and defect precursors within the smart composite material, as shown in Figure 9.

The detection system also entailed two electrodes at both ends of the composite. These electrodes were connected to an impedance analyzer, running voltage through the composite sample at 100 kHz frequency. The presence of a piezoelectric material within the composite sample will affect the collected voltage from the electrodes during mechanical testing as strain changes.

## 4. Results and Discussion

**Figure 10:**
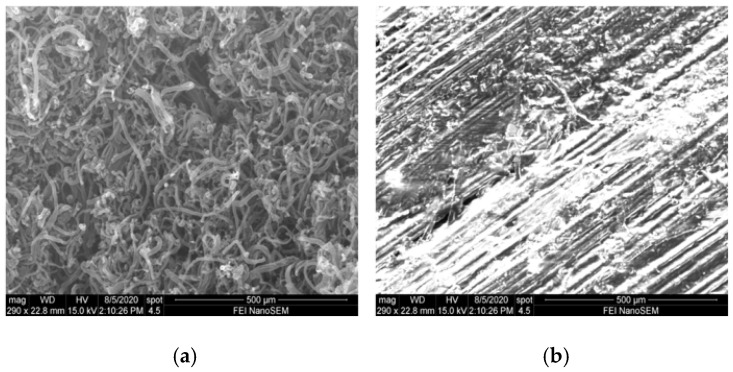
(**a**) SWCNT forest and (**b**) aligned SWCNT on prepreg glass fibers.

Characterization of the fabricated composite samples was conducted following different mechanical tests contributing to both piezoelectric and magnetostrictive responses for structural health monitoring. The distributed SWCNT morphology was examined using SEM working at 15 kV. Figure 10a shows the single-walled carbon nanotubes forest before dispersion on fibers. Prepreg fibers coated with SWCNTs are shown in Figure 10b after SWCNT alignment. The image shows lesser agglomerations of pristine SWCNTs on prepreg fibers.

**Figure 11:**
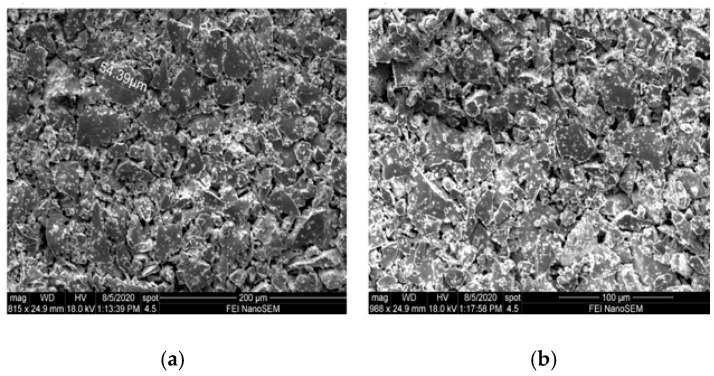
SEM images of (**a**) Terfenol-D nanoparticles before chemical treatment and (**b**) Terfenol-D nanoparticles after chemical treatment.

The variation of Terfenol-d alloy nano particles size was observed, and SEM images are shown in Figure 11, where Figure 11a shows the nanoparticles before chemical treatment and Figure 11b Terfenol-D nanoparticles after chemical treatment before dispersion on prepreg fibers. The size of the nanoparticles was found to vary from 0 to 300 µm.

**Figure 12:**
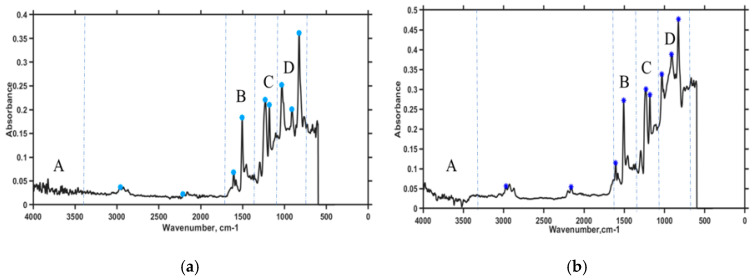
FTIR prepreg substrate SWCNT alignment characterization of samples (**a**) with no nanoparticles and (**b**) with Terfenol-D nanoparticles.

Nicolet 6700 FTIR Fourier transform infrared (FTIR) spectroscopy was used to determine the bonding characteristics of SWCNTs in prepreg samples. Thirty-two scans were collected for every sample. Several spots were targeted on the prepreg substrates coated with SWCNTs to determine FTIR absorption peaks as shown in Figure 12. Absorption measurements were performed over wavelength ranging from 600 to 4000 cm^−1^.

**Figure 13:**
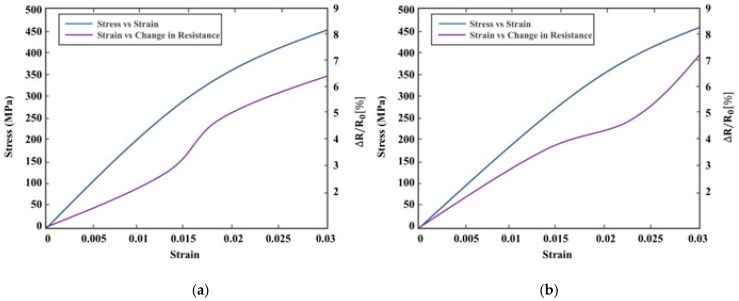
Glass fiber-reinforced SWCNTs: (**a**) non-aligned nanotubes and (**b**) aligned SWCNTs.

From FTIR analysis, it was found that the resin system in the prepreg contains diglycidyl ether of bisphenol-A mixture (DGEBA). The peak observed at 911 cm^−1^ in section D for both Figure 12a,b confirm the presence of the epoxide group, which occurs due to the stretching of C–O. However, the oxirane group peaks at 2963, 1579 and 1505 cm^−1^ (section B) represent a benzene ring which is also constituent with DGEBA (1). A slight variation in absorption peaks for prepreg with Terfenol-D (Figure 12b) was observed due to the presence of isopropanol used in chemical treatment of these nanoparticles. The presence of SWCNT can be confirmed by the peaks at 1457 and 1606 cm^−1^ which correspond to SWCNTs. The FTIR analysis shows a slight deviation when adding magnetostrictive nanoparticles to the prepreg substrate with SWCNT. From the analysis, we determined that the peak broadening in Figure 12b at the 911 and 2200 cm^−1^ (sections B, C and D) wavelength corresponds to the epoxied group and nitrile. The peak boarding may occur because of the presence of a hydrogen bond. It was found that the dipole moment intensity of the epoxied group increased, which indicates less crosslinking. Both hardener and resin undergo hydrogen bond reaction, which may cause a reduction of the C–O–C bond. Thus, Terfenol-D particles hinder the reaction between CNT and the resin system. The Terfenol-D particles also behaved like point defects or impurities in the prepreg system, which can also be a reason for peak boarding. These glass fiber-reinforced composite samples with aligned SWCNTs were passed through a series of quasi-static tensile tests to determine their overall contribution to the composite mechanical properties. The improvement of dielectric properties of the glass fiber-reinforced composite due to the presence of SWCNTs also enabled characterization of the sample’s piezoelectric response. The SWCNT weight fraction was kept constant throughout this work based on literature studies [24]. A comparison of randomly dispersed SWCNTs and aligned SWCNTs was done, and the results are shown in Figure 13.

**Figure 14:**
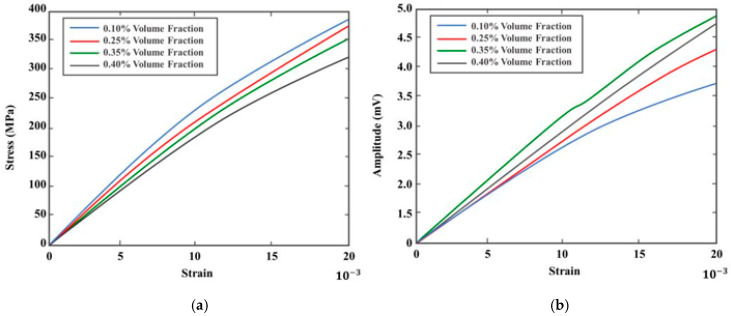
Composite with various volume fractions of SWCNTs: (**a**) stress–strain plot and (**b**) strain versus amplitude plot.

The comparison of electrical resistance change to strain was conducted for the SWCNT glass fiber-reinforced composite samples. Figure 13 shows a change in resistance for the strain results. Samples with aligned SWCNTs reached a maximum electrical resistance change of 7% as compared to non-aligned samples that increased to 6.4%. The sensitivity of non-aligned samples below strains of 0.013 was close to twice the sensitivity of aligned fibers below 0.013 strains, as shown in Figure 13a,b, respectively. A similar response was found in [25] where GFRP composite samples with sprayed-on CNT film were passed through a series of tensile tests monitoring the change in electrical resistance. Increase in tensile loading showed a linear change in electrical resistance below 0.015 strain. Minor defects generation above 0.015 strain affected the linearity of the electrical resistance change for both samples, showing a slight increase in slope and decline towards failure. This trend was also observed in [26], where piezoelectric properties of GFRP composites embedded with CNTs using different techniques were explored. Addition of Terfenol-D nanoparticles to the sample enabled the characterization of the Villari effect. This was achieved by comparing the change in voltage from the excitation coil as the magnetization around the composite sample changed with strains. The sensor dependence on the magnetic field applied is shown in the Section 3 (Experimental Methodology) and previous work [13]. Measurements were taken at room temperature using the SR860 Lock-in amplifier with AC excitation signal at 50 kHz. Sensor response repeatability was studied in [13], where the authors characterize cyclic loading of the composite sample at different loading rates. The detection device was observed to have consistent results with change in strain and generation of damage precursors. The response beyond composite failure lacked repeatability and, therefore, was not considered in this work. Linear amplitude response regression line gradient gave a standard error of 0.0035 (0.35%) with sensor resolution limited by the excitation signal and coil size. Different volume fractions of nanoparticles were studied in this work, and responses to strain were compared. Figure 14a shows the sample stress–strain responses for all volume fractions and Figure 14b the change in amplitude.

**Figure 15:**
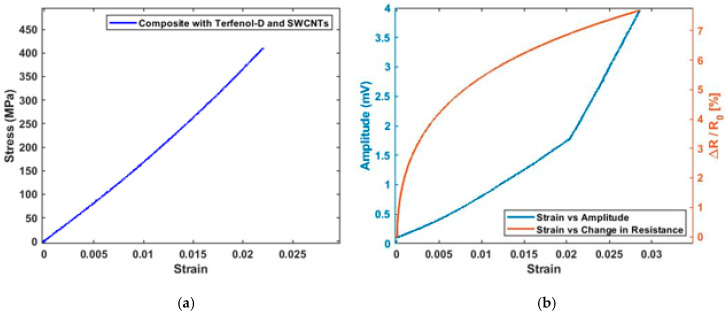
SWCNT/Terfenol-D composites: (**a**) stress–strain response; (**b**) amplitude response and change in electrical resistance.

Composite samples with 0.35% volume fraction of Terfenol-D particles showed optimal change in the supplied voltage; therefore, this constituent ratio was selected for the fabrication of the self-sensing composite. Samples with Terfenol-d nanoparticles with a volume fraction greater than 0.35% reached their ultimate stress point quicker. From Figure 15a, it can be observed that the volume fraction of 0.10% has the highest modulus of elasticity of 22.94 GPa, but Figure 15b shows the lowest change in magnetization as per the amplitude change reaching a maximum of 3.6 mV. The volume fraction of Terfenol-D nanoparticle increase reduced the composite stiffness properties. Samples with the highest volume fraction of Terfenol-D nanoparticles had the lowest modulus of elasticity—0.40% volume fraction with the lowest overall slope, as displayed in Figure 14a. A similar trend was observed by Duenas and Carman in [27], where the increase in Terfenol-D volume fraction beyond 20% volume fraction affected their composite’s stiffness properties. The increase in volume fraction of Terfenol-D also showed a direct increase in change in magnetization, which is reflected by the increase in picked-up amplitude (mV) shown in Figure 15b for samples with a volume fraction of 0.40% and 0.35%. The sample with the 0.35% volume fraction showed a higher change in amplitude, reaching its maximum at 4.8 mV, as shown in Figure 14b. This increase in amplitude with the increase in Terfenol-D nanoparticles showed the dependency of the Villari sensing properties of the self-sensing composite on the Terfenol-D volume fraction. This was also observed in [28], where an increase in Terfenol-D nanoparticles caused an increase in the magnetic polarization.

Following the optimal combinations of constituents from both piezoelectric SWCNT tests and magnetostrictive Terfenol-D tests, a final type composite with both SWCNTs and Terfenol-D nanoparticles was fabricated and went through a series of quasi static tensile tests. The tests showed a low change in Young’s modulus of the sample, as illustrated in Figure 15a,b by both the change in amplitude and electrical resistance. Both the change in amplitude and $∆R/R_{0}$(%) increased with increasing strain on the composite samples. At lower strains of 0.005, the magnetostrictive sensor showed a higher sensitivity of 290 mV/ε and gradually decreased with increasing strains. The change in the electrical resistance gradient at strains below 0.02 was low and showed an increase in sensitivity from 3.8% to sample failure at a maximum stress of 420 MPa. The difference in change in electrical resistance shown in Figure 15b to that in Figure 13b is a reflection of Terfenol-d nanoparticles contributing to the overall composite electrical resistance. 

The same linear relation at strains of 0.0017 in a composite with both SWCNTs and Terfenol-D was observed in the stress–strain plot compared to composite samples without any nanoparticles. Higher sensitivity at strains below 0.005 in the magnetostrictive sensor aligned with data from specimens that only had Terfenol-D nanoparticles, as shown in Figure 15b. This showed that the presence of SWCTs did not affect the magnetostrictive material response to strain. The experimental results for the four samples, GFRP composite, GFRP embedded with SWCNT, GFRP with Terfenol-D nanoparticles and GFRP with both SWCNT and Terfenol-D nanoparticles, were compared with COMSOL Multiphysics^®^ data for the GFRP-SWCNT-Terfenol-D system. In the COMSOL model, the applied boundary load directly affects the electrical polarization of the composite system with aligned SWCNTs. In experimental tests, the difference in change in electrical resistance in samples with SWCNTs from COMSOL Multiphysics^®^ data was due to different factors that include the uniformity of SWCNT distribution of the composite sample, partial misalignment of SWCNTs and electrical losses on electrodes connected to an impedance analyzer. The composite Villari effect in the experimental work was affected by the size of the pickup coil, the distance between the composite sample and the pickup coil and the applied magnetic field. The variation of this parameters contributed to the experimental error. This is reflected by the difference in sensitivities, as shown in Table 2. The difficulty in validation of the numerical model with experimental work was due to the differences in testing conditions of the simulation model, which include minor differences in fabricated composite samples and other environmental factors.

**Figure 16:**
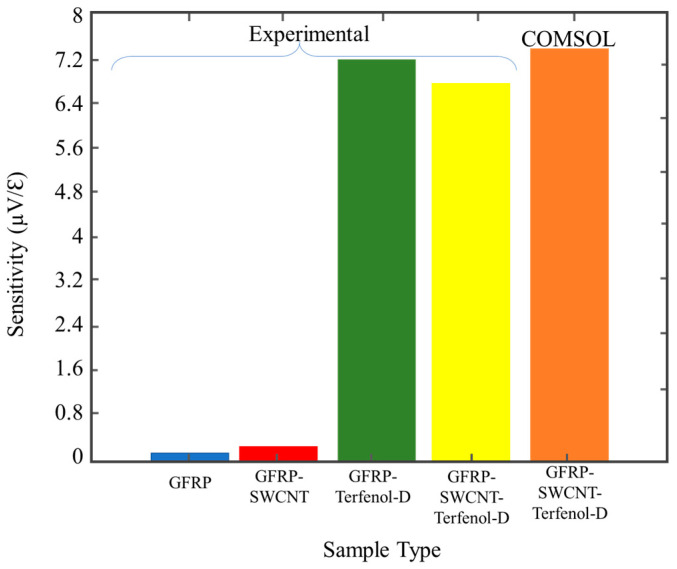
Sensor sensitivity variation for different samples in both experiment and COMSOL data.

The composite sample modelled in COMSOL Multiphysics^®^ showed to have a better sensitivity than those in experimental work with the sample concentration of Terfenol-D nanoparticles. The presence of Terfenol-D nanoparticles appeared to reduce the overall composite elastic modulus from 22.72 to 22.31 GPa. A slight increase in elastic modulus was observed with the addition of SWCNT, therefore proving to supplement the composite mechanical properties. From the experimental specimens, GFRP samples with Terfenol-D nanoparticles proved to have a higher sensitivity, which was slightly affected by the addition of SWCNTs, decreasing from 7.243 to 6.495 µV/µε, as shown in Figure 16. The multi-physics model (COMSOL) of GFRP material with SWCNT and Terfenol-D nanoparticles showed higher sensitivity. The deviation of the modelled sensitivity value from the experimental sensitivity value might be the reason for the testing setup factors contributing to experimentation error as highlighted above. 

**Figure 17:**
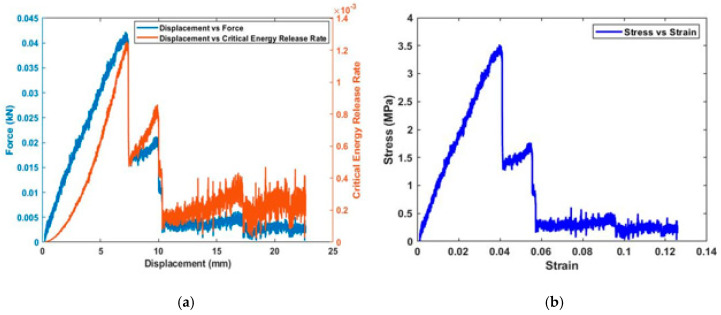
SWCNT/Terfenol-D composites: (**a**) force displacement and delamination plots; (**b**) critical strain energy release rate with change in displacement.

The next step of the tests was to determine how the fracture toughness of the composite was affected by the presence of both Terfenol-D nanoparticles and SWCNTs. This was achieved by exploring the delamination fracture toughness of the fabricated smart self-sensing composite. The resistance of the crack growth of the composite with applied strains is shown in Figure 17a, and strain energy release is shown in Figure 17b. Multiple samples with the same ratios of nanoparticles were tested and compared with samples without any nanoparticles. The crack propagation was dependent on the stress, the length of the initial crack and the geometric factor. This illustrates the relation of the stress intensity factor with fracture toughness. Crack propagation was found to be stable at low displacements of up to 5.4 mm (Figure 17a) equivalent to the strain of 0.034. Mode 1 crack is further explored through the plot, showing a drop in stress intensity factor as the crack becomes more unstable, and reduction in critical energy release rate is observed.

**Figure 18:**
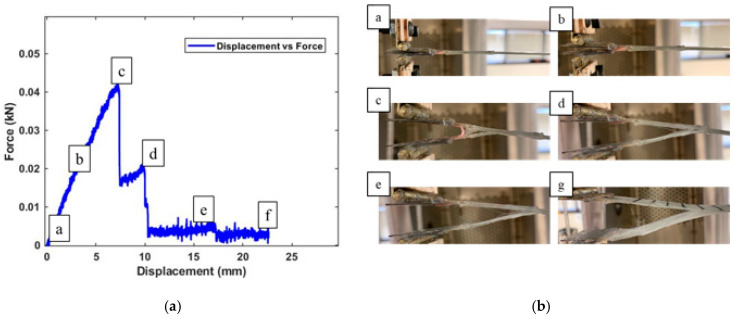
SWCNT/Terfenol-D composite: (**a**) delamination location on the displacement response curve and (**b**) crack propagation images.

To further understand the mode 1 crack propagation for the fabricated smart self-sensing composite specimens, crack images were taken as delamination continued (Figure 18a), labeling the location of where the images were taken in relation to displacement force and the propagation of cracks through the composite images (Figure 18b).

The area under the stress–strain curve up to the point of fracture at maximum stress of 3.5 MPa shown in Figure 17b represents the toughness of the composite material. This is essentially the amount of energy that is required to break these SWCNT/Terfenol-D composite samples. In Figure 18a, the initial composite response to the applied load is shown in Image a, where the crack is still stable. Increase in load force opened up the initial prefabricated crack at Image b until it reached a point of fracture at 0.043 kN. This was observed to be a higher toughness as compared to similar samples in the literature [29,30]. Even though Terfenol-D particles may lead to reduced fracture toughness, the presence of SWCNTs improved the composite material resistance to crack propagation. The reduction of critical energy release rate shown in Figure 17a can now be observed in Figure 18b in Images c and d with an unstable crack propagation. The final stages, from crack tearing to complete failure, are shown in Images e and f.

## 5. Conclusions

This work shows the development of a smart self-sensing composite. The composite piezoelectric property is achieved from the dispersion of single-walled carbon nanotubes, and the magnetostrictive property is achieved from Terfenol-D nanoparticle dispersion in the interior of the laminate.

1. A finite element analysis (FEA) study was conducted to study the mechanical, electrical and magnetic behavior of this composite to characterize its piezoelectric and magnetostrictive self-sensing responses in the presence of applied stress. The electric polarization of the piezoelectric material was found to increase rapidly with increase in strain. High piezoelectric responses were observed at sections of the composites with the highest change in length. The COMSOL Multiphysics^®^ magnetostrictive response was characterized by studying the change in magnetic field around the composite sample. The strain change in the composite sample resulted in a higher magnetization of the composite sample. This increase in magnetization is related to the solid mechanics properties of the material following the Villari effect (i.e., the change in the magnetic susceptibility of a material when subjected to a mechanical stress), therefore showing composite sensitivity to strains.
2. Experimental tensile tests of composite samples without any particles, samples with SWCNTs, samples with Terfenol-D nanoparticles and samples with both SWCNTs and Terfenol-D nanoparticles were conducted. It was observed that increase in Terfenol-D nanoparticles volume fraction increases the change in magnetization and, therefore, voltage response up to the point of saturation. The optimum change in amplitude was observed at 0.20% volume fraction of Terfenol-D nanoparticles. A constant ratio of SWCNTs was maintained, and maximum change in electrical resistance was at 7.4%.
3. Fracture toughness for the samples with all nanoparticles was explored, and the results showed improved resistance to crack propagation. This is due to the presence of SWCNTs and, therefore, proved that the mechanical properties affected by the presence of Terfenol-D alloys in the composites can be offset by the dispersion of SWCNTs and still maintain the self-sensing property of the composite.

This work therefore contributes to the development of a wireless detection method for the design of smart self-sensing SWCNT/Terfenol-D glass fiber-reinforced composites.

## Figures and Tables

**Table 1:** Materials used on the COMSOL Multiphysics^®^ model and their properties.

Property	Unit	Glass Fiber	SWCNT	Terfenol-D
Density	g/cc	2.44	1.9	9.25
Modulus of Elasticity	GPa	72.4	34.65	50–90
Thermal Conductivity	W/(m-K)	1.3	3500	13.5
Electrical conductivity	S/m	1.05 × 10^−4^	10^6^–10^7^	1.6667 × 10^6^
Poisson’s ratio	-	0.20	0.311	0.5
Relative Permeability	-	1–4.5	100.3	2–10
Linear Magnetostriction	ppm	-	-	800–1200

**Table 2:** Comparison of experimental sensor sensitivity with COMSOL Multiphysics^®^ data.

Specimen	Peak Stress (MPa)	Young’s Modulus (GPa)	Sensitivity (µV/µε)
GFRP Experimental	370	22.72	0.0457
GFRP/SWCNT Experimental	385	22.85	0.0543
GFRP/Terfenol-D Experimental	361	22.31	7.243
GFRP/SWCNT/Terfenol-D Experimental	366	22.51	6.495
GFRP/SWCNT/Terfenol-D COMSOL	450	23.012	7.35
